# Electrochemical Bottom-Up Synthesis of Chiral Carbon Dots from L-Proline and Their Application as Nano-Organocatalysts in a Stereoselective Aldol Reaction

**DOI:** 10.3390/molecules27165150

**Published:** 2022-08-12

**Authors:** Martina Bortolami, Ingrid Izabela Bogles, Cecilia Bombelli, Fabiana Pandolfi, Marta Feroci, Fabrizio Vetica

**Affiliations:** 1 Department of Basic and Applied Sciences for Engineering (SBAI), Sapienza University of Rome, via Castro Laurenziano, 7, 00161 Rome, Italy; 2 CNR—Institute for Biological Systems, Sede Secondaria di Roma-Meccanismi di Reazione, c/o Università La Sapienza, 00185 Rome, Italy; 3 Department of Chemistry, Sapienza University of Rome, piazzale Aldo Moro, 5, 00185 Rome, Italy

**Keywords:** chiral carbon dots, nano-organocatalysts, electro-organic chemistry, sustainable chemistry, stereoselective synthesis

## Abstract

Chirality is undoubtedly a fundamental property of nature since the different interactions of optically active molecules in a chiral environment are essential for numerous applications. Thus, in the field of asymmetric synthesis, the search for efficient, sustainable, cost-effective and recyclable chiral catalysts is still the main challenge in organic chemistry. The field of carbon dots (CDs) has experienced tremendous development in the last 15 years, including their applications as achiral catalysts. Thus, understanding the implications of chirality in CDs chemistry could be of utmost importance to achieving sustainable and biocompatible chiral nanocatalysts. An efficient and cost-effective electrochemical synthetic methodology for the synthesis of L-Proline-based chiral carbon dots (CCDs) and EtOH-derived L-Proline-based chiral carbon dots (CCDs) is herein reported. The electrochemical set-up and reaction conditions have been thoroughly optimised and their effects on CCDs size, photoluminescence, as well as catalytic activity have been investigated. The obtained CCDs have been successfully employed to catalyze an asymmetric aldol reaction, showing excellent results in terms of yield, diastereo- and enantioselectivity. Moreover, the sustainable nature of the CCDs was demonstrated by recycling the catalysts for up to 3 cycles without any loss of reactivity or stereoselectivity.

## 1. Introduction

**Scheme 1:**
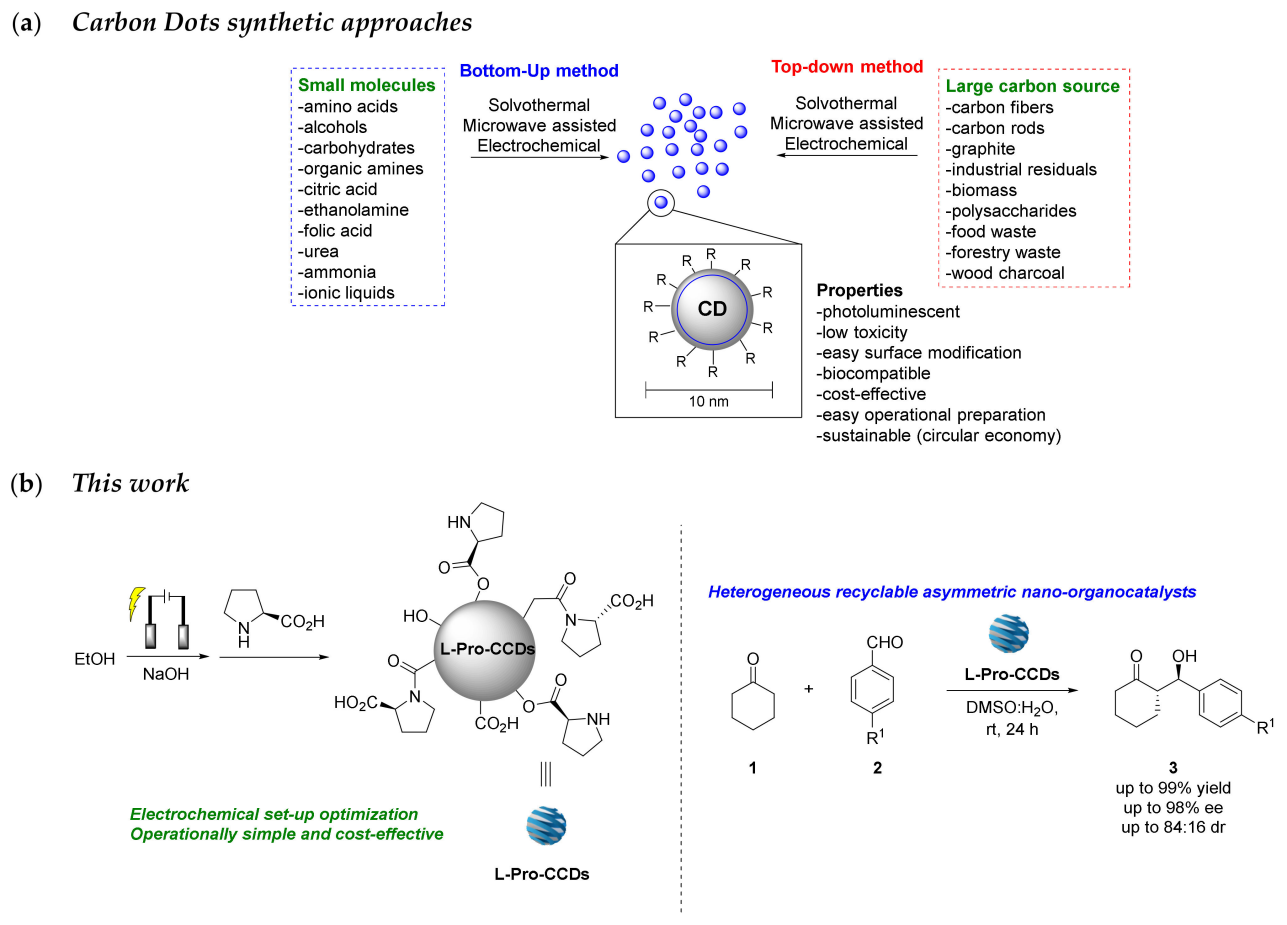
Overview of the synthetic approaches towards carbon dots: (**a**) State of the art; (**b**) this work.

In the last two decades, carbon quantum dots (CQDs), often called simply carbon dots (CDs), have emerged as one of the most important classes of the carbon-based nanoparticles family. Indeed, CDs are spherical carbon nanoparticles, with sizes ranging up to 10 nm in diameter, possessing the following plethora of peculiar properties: excellent photoluminescence with high quantum yields, good photostability, high chemical inertness and excellent dispersibility in both aqueous and organic media. Pairing with these already outstanding features, CDs have addressed the attention of various research areas due to their low toxicity, excellent biocompatibility and easy surface modification. For these reasons, CDs have found applications for in vivo imaging, fluorescence sensing, energy conversion, drug delivery and also as heterogeneous catalytic nanomaterials [1,2,3,4,5]. Additionally, the concept of sustainable chemistry and circular economy can fit the chemistry of CDs. In fact, in the nowadays always growing need for sustainable and environmentally friendly chemical processes, the easy synthetic preparation of CDs finds its place in the field of green chemistry. The two main approaches for CDs synthesis are the following: the bottom-up methods, starting from small organic molecules and the top-down approach, employing large carbon-based materials, including biomass waste (Scheme 1a) [6,7]. Moreover, the choice of starting materials and synthetic method influences the main functional groups present on the CDs’ surface. Both synthetic methods possess distinctive benefits and drawbacks, influencing in different ways the CDs’ size and photoluminescence. Nevertheless, both approaches are characterised by simple operations and cost-effective and eco-friendly techniques such as solvothermal, microwave-assisted or electrochemical treatments [8]. In the realm of CDs chemistry, the chirality aspects of these nanostructures have been less explored. It has been demonstrated by some research groups that the possibility of achieving chiral carbon dots (CCDs) via either late-stage surface modifications or with one-pot preparations starting from chiral molecules [9,10,11,12]. In fact, the chiral information of the starting material could be retained during the formation of the spherical core by being predominantly present on the active surface.

In this context, since CDs have found efficient applications as catalytic materials in organic synthesis, CCDs could represent new recyclable heterogeneous organocatalysts to promote stereoselective reactions [13,14,15]. Organocatalysis is considered the third pillar of asymmetric synthesis and, due to the green aspects of organocatalytic procedures, has paved the way for the extension of organic stereoselective synthesis horizons toward efficient, practical and more sustainable methodologies [16,17,18,19,20,21,22,23,24]. Only in the last three years, the chirality of CDs has been preliminary investigated, in four articles, for the use of these nanoparticles as heterogeneous stereoselective catalysts, showing promising results [13,25,26,27]. In particular, Xu and co-workers prepared CCDs starting from D-proline and citric acid via thermal treatment at 180 °C in the autoclave, screening the different synthetic conditions to ensure good catalytic performance of CCDs [27].

Nevertheless, in the realm of CDs synthetic approaches, electrochemical methods have shown superior control over the prepared CDs features, ensuring precise parameter optimisation. Indeed, electrochemistry can be a powerful technique in organic chemistry as it allows for heterogeneous redox reactions in which the driving force can be easily modulated by changing the potential between two electrodes. Moreover, as the redox reagent is the electron, which is cheap, intrinsically non-pollutant and easily dosable, electrochemistry can be considered a “green” way to carry out redox reactions [28,29,30].

Within this context, electrochemical methods have been proven efficient for the synthesis of CDs and very recently have shown promising results in the preparation of chiral CDs in cost-effective and operationally simple procedures [31,32,33]; however, the potential of this approach has just begun to be explored. Moreover, electrochemically derived CCDs have never been tested for their nano-organocatalytic reactivity. Due to the above excellent potentialities of CCDs, systematic screening of electrochemical synthesis of chiral CDs and its influence on the performance of CCDs as catalytic materials is still lacking.

Due to our experience in asymmetric organocatalytic methodologies [34,35,36,37,38,39] as well as in the development of innovative electrochemical synthetic methodologies [40,41,42,43,44,45], we envisioned the combination of electrochemical synthesis of CCDs to be used as sustainable, green and biocompatible nano-organocatalysts to promote stereoselective reactions (Scheme 1b). We decided to decorate the CDs with L-proline as this amino acid is frequently used in asymmetric organocatalysis.

## 2. Results and Discussion

### 2.1. Optimisation of the Electrochemical CCDs Synthesis and Purification

Our investigation started with the evaluation of the appropriate electrochemical (EC) set-up for the preparation of CCDs starting directly from L-Proline. The following two methodologies are possible: top-down [46], i.e., anodic exfoliation/decomposition of large carbonaceous materials and bottom-up, i.e., electropolymerization/carbonization of small molecules. As the majority of papers report the synthesis of CDs by a top-down approach, based on literature findings [32] we initially tested this approach by employing an alkaline-assisted electro-polymerization using two graphite rod electrodes in the presence of L-Proline. This method involves the gradual degradation of the carbon-based anode to generate the nanoparticles’ carbon cores. Simultaneously, during the electrolysis, the amino acid can functionalize in one step on the CDs active surface, leading to chiral carbon dots. The electrochemical setup consisted of a one-compartment cell working under galvanostatic conditions with a constant current of 15, 20 or 50 mA (Table 1, **CCDs-1** to **3**). The resulting slurry solutions were then filtered to remove large particles, dialyzed against pure water to remove too small particles and dried *in vacuo*. The final solid product was isolated with only poor yield; however, it was characterised by a combination of methods, indeed confirming the presence of CDs (see Section 2.2 for characterization details). The poor yield is probably due to the quite high number of large graphite particles derived from the electrode exfoliation, which are eliminated during the filtration.

Prompted by these promising initial results, we envisioned a thorough screening and combination of other different electrochemical methods to improve the preparation of these nanoparticles. In fact, this top-down approach involves the degradation of the electrode, which is not entirely desirable in a large-scale or sustainable synthetic methodology. During the last decade, a few papers were published reporting the bottom-up electrochemical synthesis of CDs, starting from alcohols [47,48,49,50], nitriles [51], sodium citrate [52], diamines [53,54] and amino acids [33,55,56]. Thus, we moved to a bottom-up approach, using ammonia-catalyzed electro-polymerization [33] of L-Proline, which serves as both a carbon-precursor and chiral template. The L-Pro electropolymerization/carbonization was carried out using the following three different electrochemical techniques: galvanostatic, potentiostatic (with a reference electrode) and under a constant potential difference (without a reference electrode). Moreover, the electrolysis time (thus, the number of Coulombs) and the kind of purification process were also changed. In fact, the amount and dimensions of the chiral nanocatalysts obtained depend on a combination of synthesis and purification steps [33,57].

When carrying out the electrolysis of an aqueous solution of L-Pro (in alkaline conditions), the cathodic reaction is hydrogen evolution, while the anodic one is the polymerization/carbonization of the organic substrate. The electrolyses were usually carried out till the brown colour of the solution or oxygen evolution at the anode. At first, potentiostatic conditions were applied (Table 1, **CCD-4** and **5**), using a three-electrode cell and purifying the resulting solution by dialysis (MWCO 1 kD, as reported in the literature [58]), but very low amounts of CDs were obtained. A similar result was obtained by carrying out the electrolyses under galvanostatic conditions (150 mA, Table 1, **CCD-6** to **8**). In all cases, quite large carbon nanomaterials were obtained (as shown by SEM measurements, not reported), while the expected CDs were found in the dialysis wastewaters. These results confirmed that the dialysis process with a membrane with 1 kDa as a cutoff was not suitable for our nanoparticles. We thus tried other methods to purify the CDs, using a membrane with a 0.5 kDa cutoff or by washing with ethanol followed by centrifugation. Indeed, better results were obtained under galvanostatic conditions and using a membrane with a 0.5 kDa cutoff for dialysis (**CCD-9**). Then, we carried out the electrochemical CDs synthesis under a constant potential difference (two electrodes), applying a ΔE of 6 to 12 V (Table 1, **CCD-10** to **14**). Low yields were obtained using a ΔE = 12 V, using a 1 kDa dialysis membrane or washing with ethanol and centrifugation (**CCD-10** and **CCD-11**, respectively), as too large particles were obtained, filtered off in the first purification step (filtration). The yields increased with decreasing ΔE, washing with ethanol and centrifugation (Table 1, **CCD-12** to **14**).

Lastly, we decided to investigate an additional bottom-up two-step method. The chiral information in CCDs is mainly due to the presence of proline residues at the active surface, while the graphite-like core is achiral. Despite the low cost and impact of using L-Proline as both a carbon source and a chiral template, most of the chiral substrate is combusted and transformed into an achiral nanoparticle core. Thus, we decided to switch to a different carbon source for the EC core generation, followed by a surface functionalization using L-Proline [47,59]. Ethanol was selected as the model substrate for the following two main reasons: (1) it is an environmentally green solvent; (2) the use of ethanol will lead to a diversely functionalized active surface. In fact, in all the previously tested cases, the use of ammonia yielded N-doped carbon dots, in which the active surface is mainly constituted by NH_2_ groups and L-Proline residues. Keeping in mind that the envisaged catalytic target of the prepared CCDs uses the classical enamine activation mode promoted by the secondary amine of proline, the presence of other amino groups could possibly interfere and lead to lower stereocontrol. Thus, using EtOH as a substrate for the CDs preparation will furnish an OH-based active surface, which could be then functionalized with L-Proline, introducing the catalytic moiety and the chiral information. An alkaline ethanol solution was electrolysed under potentiostatic conditions (+3 V anodic potential) for 5 h, yielding a new set of CDs (Table 1, **CD-15**). 

Afterwards, we tested the following two different reaction conditions for the functionalization of **CD-15** with L-Proline: adsorption and acid-catalysed esterification (Table 2). Both techniques afforded good amounts of CCDs which were characterised and added to the library to test their catalytic activity in organocatalytic reactions. 

### 2.2. Chiral CDs Characterization

**Figure 1:**
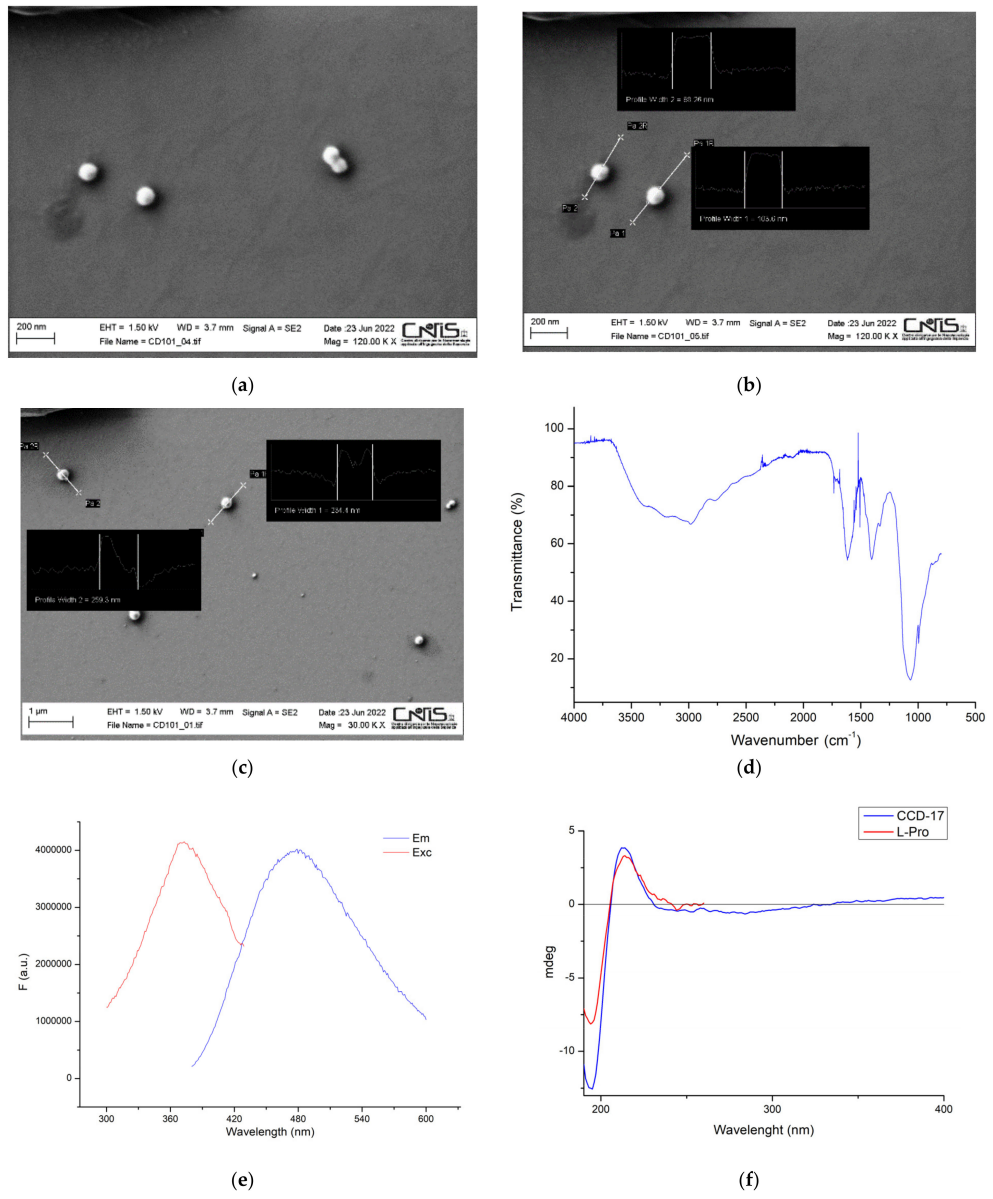
(**a–c**) Scanning electron microscopy (SEM) images of the electrochemically synthesised **CCD-17**; (**d**) IR spectrum of **CCD-17**; (**e**) Fluorescence excitation (red line) and emission (blue line) spectra of **CCD-17** (2 mg/mL); (**f**) Circular dichroism spectra of L-Pro (0.3 mM) and **CCD-17** (1 mg/mL) (red and blue line, respectively).

The electrogenerated CCDs were characterised initially via Scanning Electron Microscopy (SEM) and by fluorescence measurements (Figure 1a–c and e, respectively). The prepared chiral CDs derived exclusively from L-Proline (**CCDs-1** to **14**) exhibited fluorescent properties with a maximum excitation wavelength of ca. 340 nm and maximum emission at 440 nm, in accordance with other proline-containing carbon dots reported in the literature [27]. Similarly, the EtOH-derived functionalized with L-Pro **CCD-16** and **CCD-17** showed a maximum excitation wavelength of ca. 340 nm and maximum emission of 440 nm. 

All the obtained CCDs showed good suspensibility in both water and DMSO. The best results in terms of dimension, shape and polydispersion were obtained with **CCD-17**, for which the characterization is shown in Figure 1. The shown SEM images of the CCDs are taken under a 200 nm ruler. The images depict that CCDs are uniformly dispersed and that the average size is 171.4 nm, with a minimum of 88.0 nm to a maximum of 259.3 nm. Together with the SEM image collection, an energy dispersive X-ray (EDX) analysis was performed as well, in order to identify the principal elemental compositions of our electrogenerated CCDs. The measurements were taken from two different surface areas, and they highlighted that in the CCDs, ca. 89% of the elemental concentration as weight percentage and ca. 93% of the atomic concentration are constituted by carbon, nitrogen and oxygen (see Supporting Information for details). These data confirm the presence of proline residues at the surface of the carbon dots, which is crucial for the catalytic performance of nano-organocatalysts.

To complete the CCDs characterization, we registered IR as well as circular dichroism spectra. The IR spectrum highlights the presence of a broad band between 3000 and 3500 cm^−1^, ascribable to the free OH groups, together with peaks around 1500–1600 cm^−1^ features of C=O bonds. All the prepared CCDs exhibited a peak of interaction with the circularly polarized light, confirming the retention of the chiral information from the substrate to the obtained nanoparticles, which were then used to test their stereoselective catalytic activity.

### 2.3. Application of CCDs as Nano-Organocatalysts

With the library of CCDs in hand, we focused on the evaluation of their catalytic activity in a water/DMSO solvent system. Thus, we selected cyclohexanone (**1**) and 4-nitrobenzaldehyde (**2**) as model substrates to undergo a stereoselective aldol reaction. In fact, L-Pro is an efficient catalyst for the enamine-activation of carbonyl groups for nucleophilic additions, and, possessing the CCDs an active surface constituted by proline scaffolds, we envisioned this application. We started our investigation by carrying out some control experiments, performing the reaction in the presence of L-Pro (20 mol%), in order to compare the catalytic performances of CCDs (Table 3, entry 1). Additionally, the comparison with literature data can be made considering entries 2 and 3, and in particular, the direct comparison should be performed considering entry 3, as these solvothermal CCDs are obtained using only proline as a starting material, analogous to our work [27]. All the prepared CCDs shown in Table 1 and Table 2 were tested, however, we report in Table 3 only the experiments yielding at least trace amounts of products. We started by testing the CCDs prepared to start directly from L-Proline. Initially, the CCDs synthesised employing the top-down method (**CCD-2**) were tested, obtaining the aldol product with 60 and 69% yield, respectively, despite no stereoselectivity observed (entries 4–5). Later, we compared the outcomes using the CCDs prepared under potentiostatic (**CCD-4**) and galvanostatic (**CCD-7**) conditions with the ones obtained applying a difference of potential of 12, 10 and 8 V (**CCD-10**, **CCD-12** and **CCD-13**, respectively). 

The best results were obtained with the latter, where the highest enantomeric excess (*ee*) of 50% was observed using **CCD-12**, despite a very low yield, even after prolonging the reaction time up to 168 h (Table 3, entry 11–12). The best overall results obtained with CCDs derived solely from L-Pro were obtained using **CCD-13** (Table 3, entries 14–16). In the first trial the desired product was achieved in 60% yield after 72 h, with moderate diastereo- and enantioselectivity (59/41 *anti/syn*, 40% ee). With these results in hand, we decided to investigate the possible recycling of **CCD-13**; however, both yield and stereocontrol dramatically decreased after the first cycle. 

With the aim of improving the outcomes in terms of yield, we evaluated the presence of an acidic additive, in order to favour the enamine activation, and also the use of two differently purified CCDs. In fact, **CCD-7** and **CCD-9** were both prepared under galvanostatic conditions and purified via dialysis but using two dialysis membranes with different MWCO. Hence, we envisioned that the differences in size could influence the suspensibility and, therefore, the catalytic activity. Indeed, the CCDs obtained with the smaller size-membrane (**CCD-9**) showed a dramatic increase of yield up to 85% compared to the 22% of entry 18, probably due to the more uniform suspensibility of these smaller particles and to the higher number of CDs.

Although these previous results were promising, the diastereo- and enantioselectivity were only moderate and could not be improved simply by changing the electrochemical set-up. Thus, we decided to test the EtOH-derived CDs functionalized with L-Pro. To our delight, both showed remarkable catalytic efficiency in terms of yield and stereocontrol in 24 h (Table 3, entries 20 and 23). In particular, **CCD-16** afforded the desired product with a 94% yield, an 84/16 *anti*/*syn* diastereomeric ratio and an excellent *ee* value of 98%. Similar excellent results were obtained employing **CCD-17** (83% yield, 83/17 *anti/syn*, 96% *ee*). 

Afterwards, we decided to test the possibility of recycling these two catalysts. The recycling of **CCD-16** resulted in an unsuccessful result, with a gradual decrease in both yield and stereoselectivity (Table 3, entries 21–22), probably due to the gradual desorption of L-Proline during the subsequent extraction workups. Remarkable results were obtained with **CCD-17**, where the catalyst could be recycled up to two times without catalytic activity loss (Table 3, entries 24–25).

Prompted by these results in the DMSO:water solvent system, we also performed the reaction using **CCD-17** in pure water together with the control experiment with only L-Pro as a catalyst, however, in both cases we did not observe any results. Thus, we selected the combination of **CCD-17** in DMSO:H_2_O as the best catalytic system for the reaction in the study. 

**Scheme 2:**
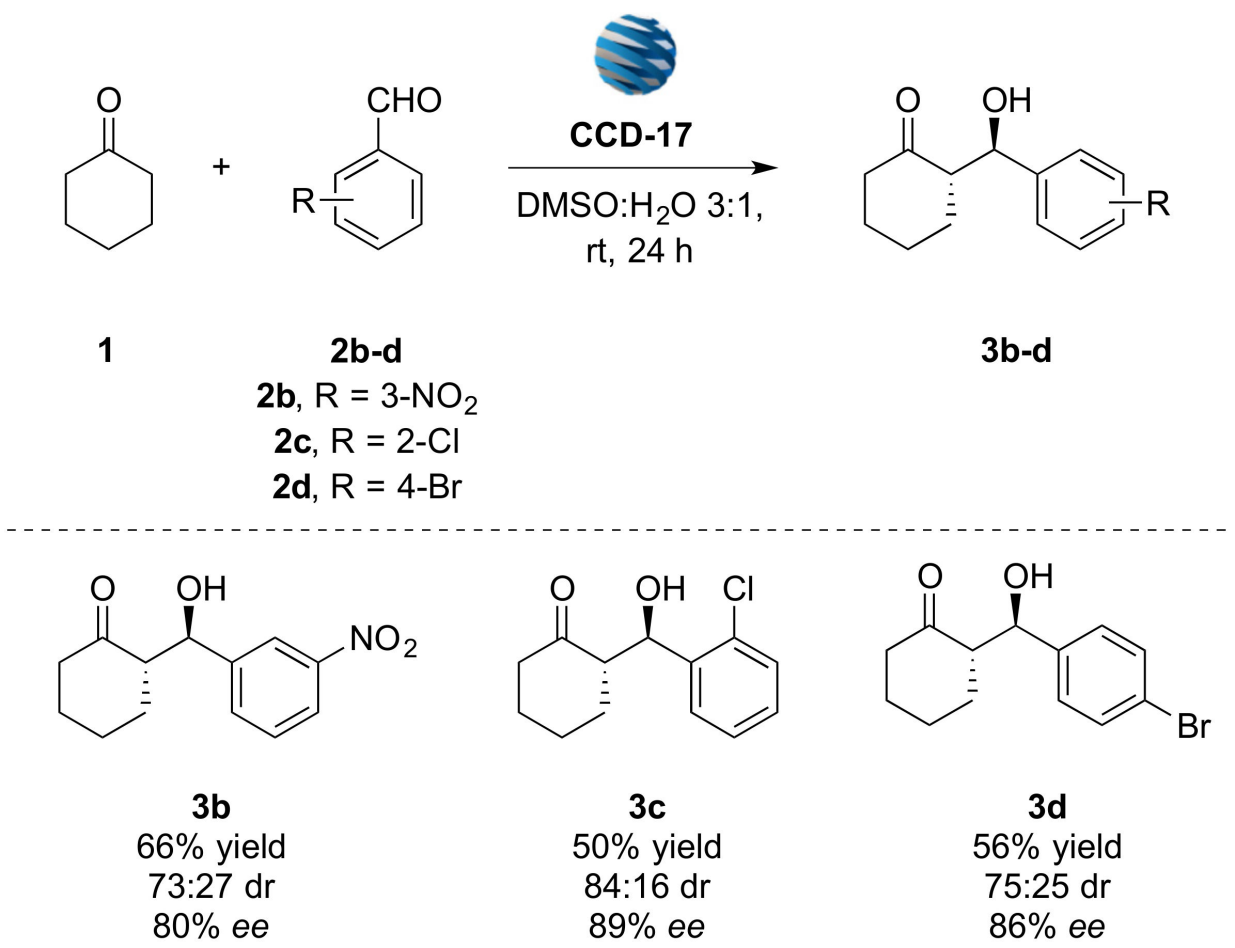
Substituent effect for the stereoselective aldol reaction catalyzed by **CCD-17**.

With the optimised conditions in hand, as proof of concept, we proceeded our study by applying our protocol to some differently substituted aldehydes in an analogous reaction system, to obtain preliminary insights on the possible influence of the substitution on the reaction outcomes. The results are summarised in Scheme 2. A general decrease in terms of isolated yields was observed in all the tested cases. This could be ascribable to the strong EWG effect of the NO_2_ group in the *para* position, as compared to, for instance, if in *meta* (**3b**). Nevertheless, in all the tested cases, similar good to excellent results were obtained considering both diastereo- and enantioselectivity. Indeed, diastereomeric ratio (dr) values ranging from 73:27 to 84:16 were observed with *ee* values between 80% and 89%, showing good functional group tolerance for these three initial substrates and promising applications towards a broader library of products. 

## 3. Materials and Methods

All chemicals were commercial (Fluorochem, Hadfield, Derbyshire, United Kingdom, and Merck, Darmstadt, Germany) and used without further purification. NMR spectra were recorded at ambient temperature on Bruker spectrometers operating at 400 MHz, or on Spinsolve 60 spectrometers operating at 60 MHz using the solvent as internal standard. The chemical shifts (δ) are given in ppm relative to TMS. Column chromatographies and flash chromatographies were carried out using silica (Merck; 63–200 or 40–63 µm particle size). Analytical HPLC was performed on a Shimadzu LC-20AD HPLC instrument equipped with a PDA detector (Shimadzu SPD-M20A) and a refractive index detector (Shimadzu RID-20A), using chiral stationary phases (Chiralpak IA, IB, IC). Fluorescence measurements were performed with a Fluoromax-3 Horiba Jobin–Yvon fluorometer (T = 25 °C). All collected data were corrected by means of a built-in program in order to counterbalance the decay in sensitivity in the near-infrared region and divided by the corrected reference detector. SEM and EDX analysis have been performed with High-Resolution Field Emission Scanning Electron Microscope (HR-FESEM) AURIGA Zeiss. Circular Dichroism spectra were recorded using JASCO J 715 spectropolarimeter with CCDs water suspensions. IR spectra were recorded with a Shimadzu FTIR-8400S spectrophotometer. Dialysis purifications were carried out with cellulose Spectra/Por^®^7 pre-treated regenerated cellulose dialysis membranes (MWCO 1 kD) or with Spectra/Por^®^ Biotech cellulose ester membranes (MWCO 0.1–0.5 kD) against distilled water. Electrolyses were performed using an Amel Model 552 potentiostat equipped with an Amel Model 731 integrator or using a K.E.R.T. Mod. K AT 4 VD stabilized power supply. The centrifugation systems used were an ALC Centrifugette 4206 and an ALC Centrifuge 4222 MKII. Sonication of the carbon dots solutions was performed using a Branson 3200 Ultrasonic Cleaner Waterbath.

### 3.1. Experimental Procedures for the Synthesis of CCDs

#### 3.1.1. Top-Down Electrochemical Synthesis

To a beaker, equipped with two parallel graphite rods (0.7 cm diameter, 1.5 cm immersed in the solution) and a magnetic stirrer, 25 (or 10) mL of water containing 200 mg (5.00 mmol) of NaOH and 250 mg (2.17 mmol) of L-Proline were added, under nitrogen atmosphere. The electrolysis, under galvanostatic conditions (15 or 20 mA, 9.4 or 12.5 mA/cm^2^ respectively) was carried out for the time reported in Table 1. The obtained CDs were then subjected to purification as reported below. When the same electrolysis was carried out under air, no difference in products was evinced and all the subsequent syntheses were carried out in air. 

When the cathode was a platinum one (flat spiral, apparent area: 1 cm^2^), 5 mL of NH_3_ 3 M in water containing 200 mg (1.74 mmol) of L-Proline were electrolysed at a constant current of 50 mA (125 mA/cm^2^) for 4.5 h. The obtained CDs were then subjected to purification as reported below.

#### 3.1.2. Bottom-Up Electrochemical Synthesis under Potentiostatic Conditions

In a beaker, equipped with two platinum electrodes (flat spirals, apparent area: 1 cm^2^), an Ag/AgCl reference electrode and a magnetic stirrer, 10 mL of NH_3_ 3 M in water containing 575 mg (5.00 mmol) of L-Proline were electrolysed under potentiostatic conditions (+3 V, anode potential value) for the time reported in Table 1. The obtained CDs were then subjected to purification as reported below.

#### 3.1.3. Bottom-Up Electrochemical Synthesis under Constant Potential Difference Conditions

In a beaker, equipped with two platinum electrodes (flat spirals, apparent area: 1 cm^2^) and a magnetic stirrer, 5 mL of NH_3_ 3 M in water containing 200 mg (1.74 mmol) of L-Proline were electrolysed under a constant potential difference (6–12 V) for the time reported in Table 1. The obtained CDs were then subjected to purification as reported below.

#### 3.1.4. Bottom-Up Electrochemical Synthesis under Galvanostatic Conditions

In a beaker, equipped with two platinum electrodes (flat spirals, apparent area: 1 cm^2^) and a magnetic stirrer, 5 mL of NH_3_ 3 M in water containing 288 (2.5 mmol) or 200 mg (1.74 mmol) of L-Proline were electrolysed under a constant current of 150 mA (150 mA/cm^2^) for the time reported in Table 1. The obtained CDs were then subjected to purification as reported below.

#### 3.1.5. Bottom-Up Electrochemical Synthesis under Potentiostatic Conditions from Ethanol

In a beaker, equipped with two platinum electrodes (flat spirals, apparent area: 1 cm^2^), a saturated calomel reference electrode (SCE) and a magnetic stirrer, 10 mL of ethanol (CH_3_CH_2_OH) mixed with a solution of 110 mg (2.75 mmol) of NaOH in 1 mL of water was electrolyzed under potentiostatic conditions (+3 V, anode potential value) for the time reported in Table 1 (current density 22–13 mA/cm^2^). At the end of the electrolysis, 10 mL of ethanol was added to the electrolyzed solution and the obtained mixture was left overnight to salt out the NaOH. The mixture was then centrifuged (5000 rpm, 5 min) washing the solid with EtOH (3 × 4 mL). The combined EtOH was concentrated under vacuum to about 3 mL, diluted with about 3 mL of water and dialyzed against ultrapure water (600 mL) through a dialysis membrane (MWCO 0.1–0.5 kD) for at least 48 h, changing the dialysis water after 24 h. The dialyzed solution was then filtered through a 0.2 µm filter (CPS 25 mm syringe filter, cellulose regenerated), concentered under vacuum and dried with the Smart Evaporator obtaining an orange solid.

#### 3.1.6. Functionalization of EtOH-Based CDs with L-Proline

The purified EtOH-based CDs were functionalized with L-Proline carrying out the following two different procedures: adsorption and acid-catalysed esterification.

Method A (adsorption): to a solution of 5.5 mg of EtOH-based CDs in 1 mL of water, 55 mg (0.48 mmol) of L-Proline were added. The obtained solution was sonicated in a ultrasonic bath for 30 min and then stirred for 2 h at room temperature. The reaction mixture was then dialyzed against ultrapure water (600 mL) through a dialysis membrane (MWCO 0.1–0.5 kD) for at least 48 h, changing the dialysis water after 24 h. The dialyzed solution was then concentered under vacuum and dried with the Smart Evaporator.

Method B (acid-catalysed esterification): to a solution of 5.5 mg of EtOH-based CDs in 5 mL of water, 55 mg (0.48 mmol) of L-Proline and 35 µL of H_2_SO_4_ conc. (96% w/w) were added. The solution was stirred at reflux for 2 h. Then the reaction mixture was neutralized with a saturated aqueous solution of NaHCO_3_ and dialyzed against ultrapure water (600 mL) through a dialysis membrane (MWCO 0.1–0.5 kD) for at least 48 h, changing the dialysis water after 24 h. The dialyzed solution was then concentered under vacuum and dried with the Smart Evaporator.

#### 3.1.7. CDs Purification

The electrolysis solutions were purified following three different procedures, as reported in Table 1. 

Method A: the electrolyzed solution was filtered through a 0.2 µm filter (CPS 25 mm syringe filter, cellulose regenerated) and then dialyzed against ultrapure water (600 mL) through a dialysis membrane (MWCO 1 kD) for at least 48 h, changing the dialysis water after 24 h. However, with these membranes a considerable amount of CDs (based on fluorescence spectra) was found in the dialysis water. The dialyzed solution was then concentered under vacuum and dried with the Smart Evaporator.

Method B: the electrolyzed solution was filtered through a 0.2 µm filter (CPS 25 mm syringe filter, cellulose regenerated) and then dialyzed against ultrapure water (600 mL) through a dialysis membrane (MWCO 0.1–0.5 kD) for at least 48 h, changing the dialysis water after 24 h. The dialyzed solution was then concentered under vacuum and dried with the Smart Evaporator.

Method C: the electrolyzed solution was filtered through a 0.2 µm filter (CPS 25 mm syringe filter, cellulose regenerated) and then dried at 40 °C overnight through the Smart Evaporator apparatus. To the obtained material 2 mL of EtOH was added, stirring for at least 30 min. The obtained homogeneous mixture was centrifuged at 6000 rpm for 5 min. After removing the liquor, the solid was further washed with EtOH and centrifuged three times and then dried with the Smart Evaporator.

### 3.2. General Procedure for the Asymmetric Aldol Reaction–Synthesis of (S)-2-((R)-Hydroxy(4-nitrophenyl)methyl)cyclohexan-1-one (**3a**)

To a suspension of CCDs (5–30 mg) in 0.5 mL of water, 1.5 mL of DMSO were added and the mixture was sonicated for 10 min. Afterwards, cyclohexanone (**1**, 5.0 mmol) and 4-nitrobenzaldehyde (**2**, 0.5 mmol) were added and the reaction was stirred for the indicated time at the specified temperature. Then, the mixture was diluted with 2 mL of water and extracted with AcOEt (4 × 5 mL); then the organic phase was washed with brine (3 × 20 mL), dried with Na_2_SO_4_ an., filtered and evaporated under reduced pressure. The crude was purified to afford the desired product. The water phase derived from the extraction, containing the CCDs, was concentred to 0.5 mL and used to perform subsequent reaction cycles.

Best results obtained using **CCD-17** (30 mg) as catalyst.

The product **3a** was isolated after flash chromatography on silica gel (hexane/EtOAc 7:3, 83% yield, 96% *ee*). All analytical data are in accordance with the literature report [60]. **HPLC**: Chiralpak IB, Hexane/iPrOH 95:5, 1.0 mL/min, *anti* diastereomer (major): *τ_major_* = 23.8 min *τ_minor_* = 28.2 min; *syn* diastereomer (minor): *τ_major_* = 20.0 min *τ_minor_* = 21.9 min. **dr**: *anti/syn* 83:17 (from NMR analysis of the crude mixture and confirmed by HPLC). *Anti* diastereoisomer: **^1^H NMR** (400 MHz, CDCl_3_): *δ* 8.26–8.16 (m, 2H), 7.55–7.45 (m, 2H), 4.90 (d, *J* = 8.3 Hz, 1H), 2.63–2.54 (m, 1H), 2.53–2.45 (m, 1H), 2.42–2.30 (m, 1H), 2.11 (tdd, *J* = 22.6, 12.9, 10.0 Hz, 1H), 1.89–1.78 (m, 1H), 1.75–1.48 (m, 4H), 1.45–1.30 (m, 1H) ppm. **^13^C NMR** (101 MHz, CDCl_3_) *δ* = 214.5, 148.3, 146.8, 127.9, 127.9, 126.6, 123.6, 77.3, 77.0, 76.7, 74.0, 57.2, 56.8, 42.7, 30.8, 27.6, 24.7 ppm.

#### 3.2.1. (S)-2-((R)-Hydroxy(3-nitrophenyl)methyl)cyclohexan-1-one (**3b**)

Product **3b** was prepared following the general procedure (66% yield, 80% *ee*). All analytical data are in accordance with the literature report [60]. **HPLC**: Chiralpak IA, Hexane/iPrOH 9:1, 0.6 mL/min, *anti* diastereomer (major): *τ_major_* = 32.3 min *τ_minor_* = 39.5 min. **dr**: *anti/syn* 73:27 (from NMR analysis of the crude mixture, and confirmed by HPLC). **^1^H NMR** (400 MHz, CDCl_3_): *δ* 8.21 (t, *J* = 2.0 Hz, 1H), 8.16 (ddd, *J* = 8.2, 2.3, 1.1 Hz, 1H), 7.67 (dt, *J* = 7.7, 1.1 Hz, 1H), 7.52 (t, *J* = 7.9 Hz, 1H), 4.89 (d, *J* = 8.5 Hz, 1H), 2.67–2.57 (m, 1H), 2.54–2.46 (m, 1H), 2.37 (tdd, *J* = 13.6, 6.1, 1.2 Hz, 1H), 2.11 (ddd, *J* = 11.7, 6.0, 2.8 Hz, 1H), 1.87–1.79 (m, 1H), 1.73–1.50 (m, 3H), 1.45–1.32 (m, 1H) ppm. **^13^C NMR** (101 MHz, CDCl_3_) *δ* = 214.90, 148.30, 143.25, 133.21, 133.20, 131.96, 129.32, 122.90, 122.04, 74.06, 57.14, 42.68, 42.63, 30.75, 27.64, 24.67 ppm.

#### 3.2.2. (S)-2-((R)-(2-chlorophenyl)(hydroxy)methyl)cyclohexan-1-one (**3c**)

Product **3c** was prepared following the general procedure (50% yield, 89% *ee*). All analytical data are in accordance with the literature report [60]. **HPLC**: Chiralpak IB, Hexane/iPrOH 95:5, 1.0 mL/min, *anti* diastereomer (major): *τ_major_* = 7.5 min *τ_minor_* = 8.3 min. **dr**: *anti/syn* 84:16 (from NMR analysis of the crude mixture, and confirmed by HPLC). **^1^H NMR** (400 MHz, CDCl_3_): *δ* 7.52 (dd, *J* = 7.7, 1.7 Hz, 1H), 7.32–7.25 (m, 2H), 7.21–7.15 (m, 1H), 5.33 (d, *J* = 8.2 Hz, 1H), 3.96 (bs, 1H), 2.71–2.59 (m, 1H), 2.44 (ddd, *J* = 13.6, 5.1, 3.1 Hz, 1H), 2.31 (ddd, *J* = 13.6, 7.2, 1.1 Hz, 1H), 2.05 (dtd, *J* = 8.8, 5.7, 2.8 Hz, 1H), 1.81–1.73 (m, 1H), 1.72–1.47 (m, 4H) ppm. **^13^C NMR** (101 MHz, CDCl_3_) *δ* = 215.17, 139.14, 132.93, 129.20, 129.19, 129.18, 129.17, 128.74, 128.29, 128.28, 127.26, 70.37, 67.62, 57.60, 57.59, 42.68, 30.38, 27.82, 24.87 ppm.

#### 3.2.3. (S)-2-((R)-(4-bromophenyl)(hydroxy)methyl)cyclohexan-1-one (**3d**)

Product **3d** was prepared following the general procedure (56% yield, 86% *ee*). All analytical data are in accordance with the literature report [60]. **HPLC**: Chiralpak IC, Hexane/iPrOH 95:5, 1.0 mL/min, *anti* diastereomer (major): *τ_major_* = 25.9 min *τ_minor_* = 30.6 min. **dr**: *anti/syn* 75:25 (from NMR analysis of the crude mixture, and confirmed by HPLC). **^1^H NMR** (400 MHz, CDCl_3_): *δ* 7.49–7.38 (m, 2H), 7.22–7.12 (m, 2H), 4.74 (d, *J* = 8.7 Hz, 1H), 3.75 (bs, 1H), 2.60–2.39 (m, 2H), 2.39–2.25 (m, 1H), 2.13–2.02 (m, 1H), 1.84–1.70 (m, 1H), 1.70–1.47 (m, 3H), 1.27 (ddd, *J* = 13.6, 13.1, 9.7 Hz, 1H) ppm. **^13^C NMR** (101 MHz, CDCl_3_) *δ* = 215.31, 140.15, 131.55, 131.32, 128.84, 127.65, 121.77, 74.22, 74.21, 57.41, 57.40, 42.73, 30.82, 27.81, 24.78 ppm.

## 4. Conclusions

In conclusion, a straightforward electrochemical synthesis of chiral carbon dots starting from L-Proline or simple EtOH and L-Proline has been developed, with a thorough screening of the electrochemical set-up and its influence on the nanoparticle properties as well as catalytic activity. Indeed, the obtained CCDs have been successfully applied as nano-organocatalysts to perform an aldol reaction in DMSO:H_2_O solvent system, pairing excellent stereoselectivities (up to 84:16 *anti/syn* and 98% *ee*) with the possibility to recycle the catalytic nanoparticles without reactivity loss when EtOH and L-Proline derived CDs were employed. Due to the always-growing need for sustainable and environmentally friendly processes in asymmetric synthesis, the horizons for organic synthesis have shifted to new technologies as well as the recyclability of catalytic systems. We believe that this methodology could be a promising step towards the development of a plethora of carbon-based chiral nanoparticles for their applications as heterogeneous catalytic systems, relying on the green features of a green electrochemical setup.

## Figures and Tables

**Table 1:** Reaction conditions screening for the electrochemical synthesis of CCDs from L-Proline.

CCDs	Starting Materials	Approach	H_2_O [mL]	Electrodes +/−/Ref	I/E/ΔE	Time [h]	Q [C]	F/mol	CCDs [mg]
**CCD-1** ^(a)^	L-Pro (250 mg)/NaOH (200 mg)	Top-down	25	C/C/- ^(b)^	15 mA	72	3888	18.5	5.0
**CCD-2** ^(a)^	L-Pro (250 mg)/NaOH (200 mg)	Top-down	10	C/C/- ^(b)^	20 mA	76	5472	26.1	2.1
**CCD-3** ^(c)^	L-Pro (200 mg)/NH_3_ (3 M)	Top-down	5	C/Pt/- ^(d)^	50 mA	4.6	828	4.9	2.5
**CCD-4** ^(a)^	L-Pro (575 mg)/NH_3_ (3 M)	Bottom-up	10	Pt/Pt/Ag-AgCl ^(e)^	+3 V	2	864	1.8	1.6
**CCD-5** ^(a)^	L-Pro (575 mg)/NH_3_ (3 M)	Bottom-up	10	Pt/Pt/Ag-AgCl ^(e)^	+3 V	4	1555	3.2	2.0
**CCD-6** ^(a)^	L-Pro (288 mg)/NH_3_ (3 M)	Bottom-up	5	Pt/Pt/- ^(e)^	150 mA	7	3780	15.7	2.4
**CCD-7** ^(a)^	L-Pro (200 mg)/NH_3_ (3 M)	Bottom-up	5	Pt/Pt/- ^(e)^	150 mA	3	1620	9.6	3.8
**CCD-8** ^(c)^	L-Pro (200 mg)/NH_3_ (3 M)	Bottom-up	5	Pt/Pt/- ^(e)^	150 mA	3	1620	9.6	2.6
**CCD-9** ^(f)^	L-Pro (200 mg)/NH_3_ (3 M)	Bottom-up	5	Pt/Pt/- ^(e)^	150 mA	3	1620	9.6	7.2
**CCD-10** ^(a)^	L-Pro (200 mg)/NH_3_ (3 M)	Bottom-up	5	Pt/Pt/- ^(e)^	ΔE = 12 V	0.5	825	4.9	1.7
**CCD-11** ^(c)^	L-Pro (200 mg)/NH_3_ (3 M)	Bottom-up	5	Pt/Pt/- ^(e)^	ΔE = 12 V	0.5	851	5.0	2.5
**CCD-12** ^(c)^	L-Pro (200 mg)/NH_3_ (3 M)	Bottom-up	5	Pt/Pt/- ^(e)^	ΔE = 10 V	0.7	992	5.9	4.7
**CCD-13** ^(c)^	L-Pro (200 mg)/NH_3_ (3 M)	Bottom-up	5	Pt/Pt/- ^(e)^	ΔE = 8 V	1.5	1704	10.1	22
**CCD-14** ^(c)^	L-Pro (200 mg)/NH_3_ (3 M)	Bottom-up	5	Pt/Pt/- ^(e)^	ΔE = 6 V	5	4153	24.7	46
**CD-15** ^(f)^	EtOH (10 mL)/NaOH (110 mg)	Bottom-up	1	Pt/Pt/Hg_2_Cl_2_^(e)^	+ 3 V	5	300		10

^(a)^ Purified by dialysis (MWCO 1 kD); ^(b)^ Apparent electrode surface area: 1.6 cm^2^; ^(c)^ Purified by washing with EtOH and centrifuged at 6000 rpm for 5 min; ^(d)^ Apparent electrode surface area: 0.4 cm^2^; ^(e)^ Apparent electrode surface area: 1.0 cm^2^; ^(f)^ Purified by dialysis (MWCO 100–500 D). See Supplementary Materials and Methods.

**Table 2:**
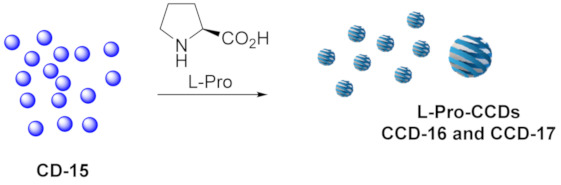
Functionalization of EtOH-based CDs (**CD-15**) with L-Proline.

CCDs	CD-15 [mg]	Approach	H_2_O [mL]	Reagents	Reaction Conditions
**CCD-16** ^(a)^	5.5	Adsorption	1	L-Pro (55 mg)	30 min sonication + 2 h stirring at rt
**CCD-17** ^(a,b)^	5.5	Functionalization	5	L-Pro (55 mg) H_2_SO_4_ conc. (35 µL)	2 h reflux

^(a)^ Purified by dialysis (MWCO 100–500 D); ^(b)^ after the elapsed time the solution was neutralised with NaHCO_3_ s.s. prior dialysis.

**Table 3:**
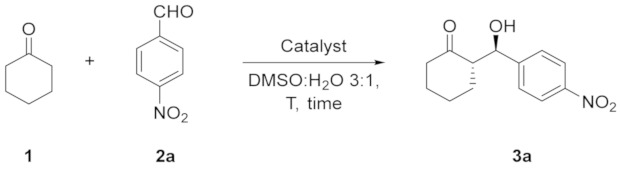
Stereoselective aldol reaction promoted by differently electrochemical synthesised CCDs.

Entry ^(a)^	Catalyst	1/2 [mmol]	T [°C] ^(b)^	Time [h]	Yield [%] ^(b)^	*anti/syn* ^(b)^	*ee* [%] ^(c)^
**1**	L-Pro (20 mol%)	5/0.5	rt	24	99	93/7	96
**2** [27]	Solvothermal CCDs (D-proline and citric acid)	5/0.5	rt	24	98	98/2	−71
**3** [27]	Solvothermal CCDs (only D-proline)	5/0.5	rt	24	58	97/3	−81
**4**	**CCD-2** (top-down)	5/0.5	rt	72	60	51/49	0
**5**	**CCD-2** (top-down)	5/0.5	rt	162	69	42/58	0
**6**	**CCD-4** (potentiostatic +3 V)	5/0.5	rt	144	traces	-	-
**7 ^(d)^**	**CCD-7** (galvanostatic 150 mA)	5/0.5	rt	24	traces	70/30	34
**8 ^(d)^**	**CCD-7**	5/0.5	40	24	7	56/44	12
**9 ^(d)^**	**CCD-7**	5/0.5	rt	168	16	68/42	12
**10**	**CCD-10** (12 V)	5/0.5	rt	168	15	74/26	40
**11 ^(d)^**	**CCD-12** (10 V)	5/0.5	rt	24	10	70/30	48
**12 ^(d)^**	**CCD-12**	5/0.5	rt	168	28	70/30	50
**13**	**CCD-13** (8V)	5/0.5	rt	24	15	70/30	42
**14** ^(d)^	**CCD-13** (8 V)	5/0.5	rt	72	60	59/41	40
**15** ^(d)^	**CCD-13**	5/0.5	rt	72	35	59/41	21
**16** ^(d)^	**CCD-13**	5/0.5	rt	72	5	59/41	-
**17** ^(e)^	**CCD-13** + benzoic acid	5/0.5	rt	96	traces	-	-
**18**	**CCD-7** (1000 MWCO dialysis)	5/0.5	rt	72	22	50/50	49
**19**	**CCD-9** (500 MWCO dialysis)	5/0.5	rt	24	85	48/52	26
**20** ^(d)^	**CCD-16** (EtOH-derived with L-Pro adsorbed)	5/0.5	rt	24	94	84/16	98
**21** ^(d)^	**CCD-16**	5/0.5	rt	24	78	78/22	80
**22** ^(d)^	**CCD-16**	5/0.5	rt	48	traces	-	-
**23** ^(d)^	**CCD-17** (EtOH-based derivatised with L-Pro)	5/0.5	rt	24	83	83/17	96
**24** ^(d)^	**CCD-17**	5/0.5	rt	24	102	84/16	98
**25** ^(d)^	**CCD-17**	5/0.5	rt	24	57	81/19	96
**26** ^(f)^	**CCD-17**	5/0.5	rt	24	0	-	-
**27** ^(f)^	L-Pro (20% mol)	5/0.5	rt	24	0	-	-

^(a)^ Reaction performed using 2 mL of a 3:1 DMSO/H_2_O mixture as solvent. Dispersion of CCDs sonicated for 10 min prior addition of the substrates to guarantee homogeneous dispersion; ^(b)^ determined via ^1^H NMR analysis of the crude; ^(c)^ determined by HPLC on a chiral stationary phase; ^(d)^ the same catalyst was recycled for the indicated reactions; ^(e)^ reaction performed in the presence of 20 mol% of benzoic acid; ^(f)^ reaction performed using only water as solvent.

## Data Availability

Not applicable.

## Supplementary Materials

The following supporting information can be downloaded at: https://www.mdpi.com/article/10.3390/molecules27165150/s1, EDX analysis, ^1^H and ^13^C NMR spectra, HPLC chromatograms.
