# Supplementary material for: Electrochemical Bottom-Up Synthesis of Chiral Carbon Dots from L-Proline and Their Application as Nano-Organocatalysts in a Stereoselective Aldol Reaction
Source: Molecules. 2022 Aug 12;27(16):5150. doi: 10.3390/molecules27165150 (PMC9414281; doi:10.3390/molecules27165150)
Supplement: Supplementary file 1 [file molecules-27-05150-s001.zip › molecules-1863106-supplementary.pdf]

Supplementary Information

Electrochemical bottom-up synthesis of Chiral Carbon Dots and their application as Nano-Organocatalysts in a stereoseletive Mannich reaction in water

Martina Bortolami,[1] Ingrid Izabela Bogles,[1] Cecilia Bombelli,[2] Fabiana Pandolfi,[1,2] Marta Feroci,[1,*] and Fabrizio Vetica[3,*]

[1]*Department of Basic and Applied Sciences for Engineering (SBAI), Sapienza University of Rome, via Castro Laurenziano, 7, 00161 Rome (Italy).*

[2]*Institute of Biological Systems (ISB), Italian National Research Council (CNR), Sezione Meccanismi di Reazione, Piazzale Aldo Moro 5, 00185 Rome, Italy.*

[3]*Department of Chemistry, Sapienza University of Rome, piazzale Aldo Moro, 5, 00185 Rome (Italy).*

Table of Contents

Energy Dispersive X-ray (EDX) analysis 2

NMR SPECTRA 3

HPLC DATA 7

Energy Dispersive X-ray (EDX) analysis

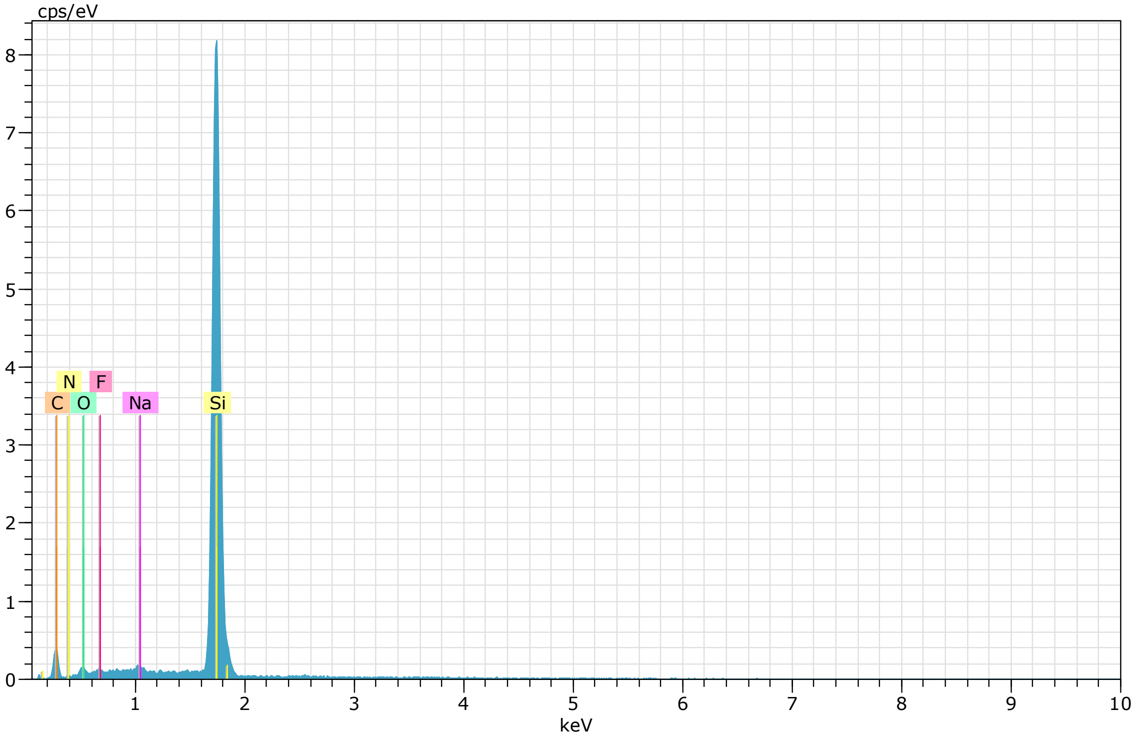

Spectrum: CD101_ 3

Element	Series	unn. C [wt.%]	norm. C [wt.%]	Atom. C [at.%]	Error (1 Sigma) [wt.%]
Silicon	K-series	0.00	0.00	0.00	0.00
Carbon	K-series	1.11	45.01	54.37	0.30
Oxygen	K-series	0.68	27.38	24.83	0.24
Fluorine	K-series	0.31	12.52	9.56	0.15
Sodium	K-series	0.27	10.89	6.87	0.05
Nitrogen	K-series	0.10	4.21	4.36	0.12
	Total:	2.47	100.00	100.00	

NMR Spectra

(S)-2-((R)-hydroxy(4-nitrophenyl)methyl)cyclohexan-1-one (3a)

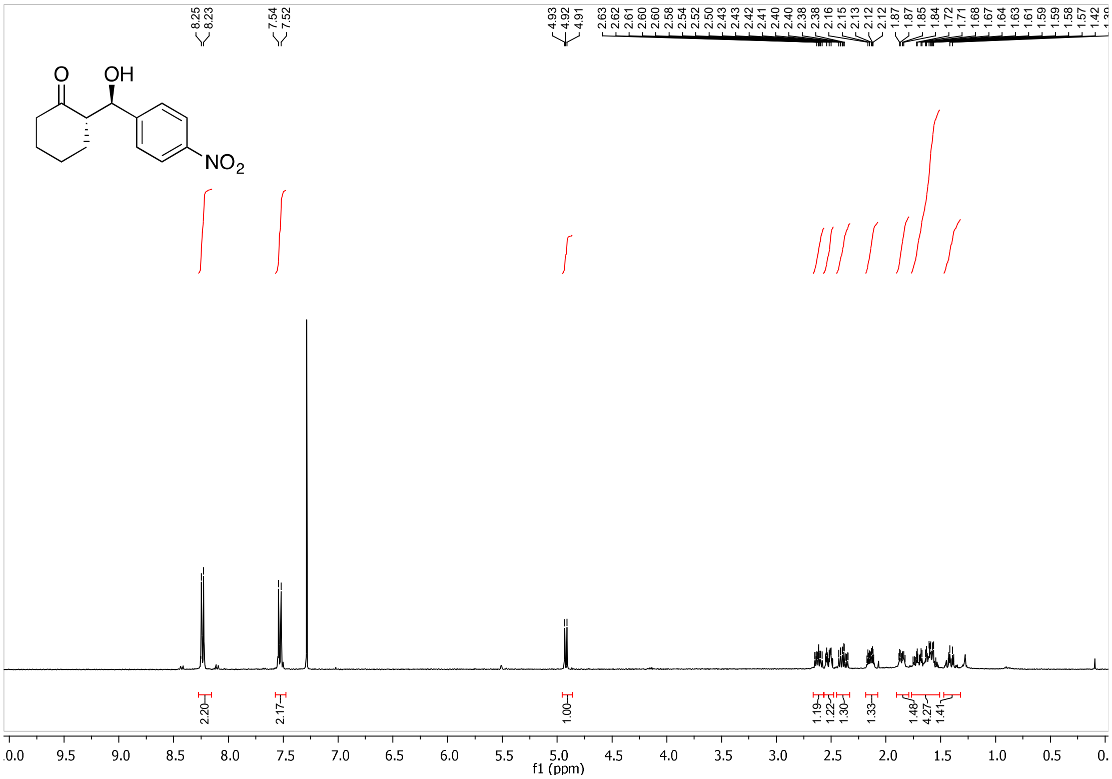

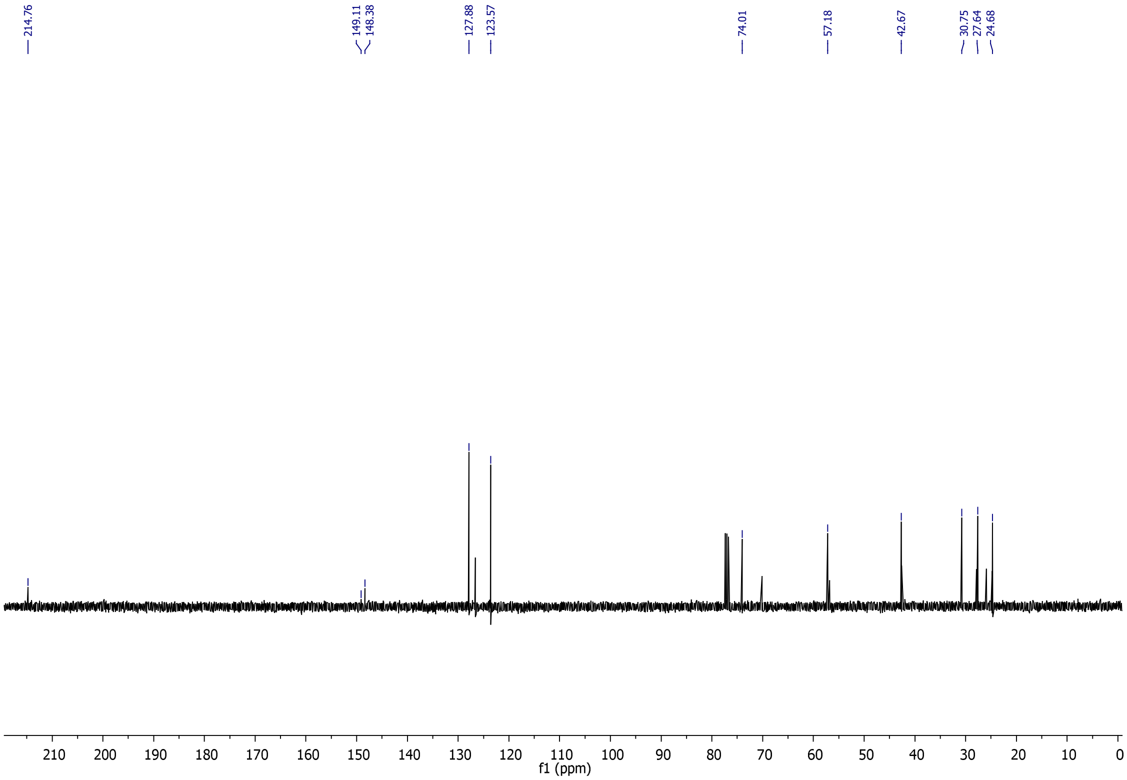

(S)-2-((R)-hydroxy(3-nitrophenyl)methyl)cyclohexan-1-one (3b)

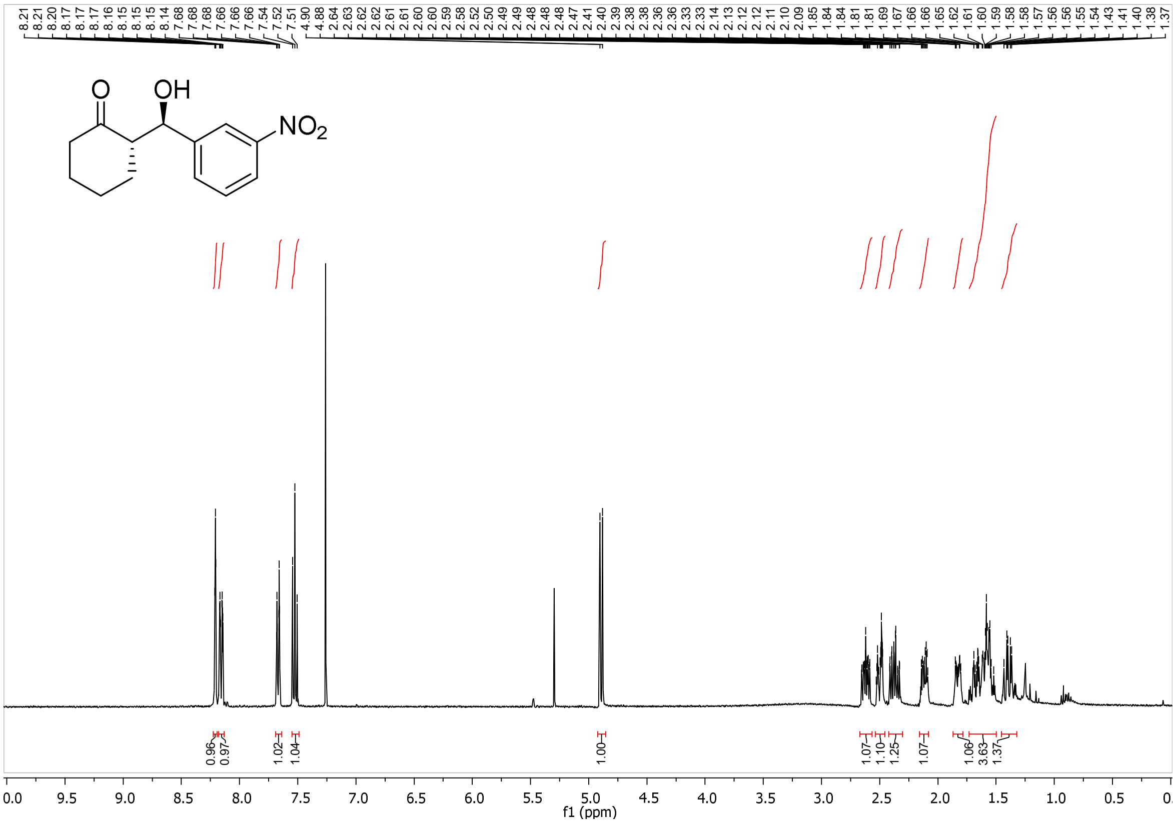

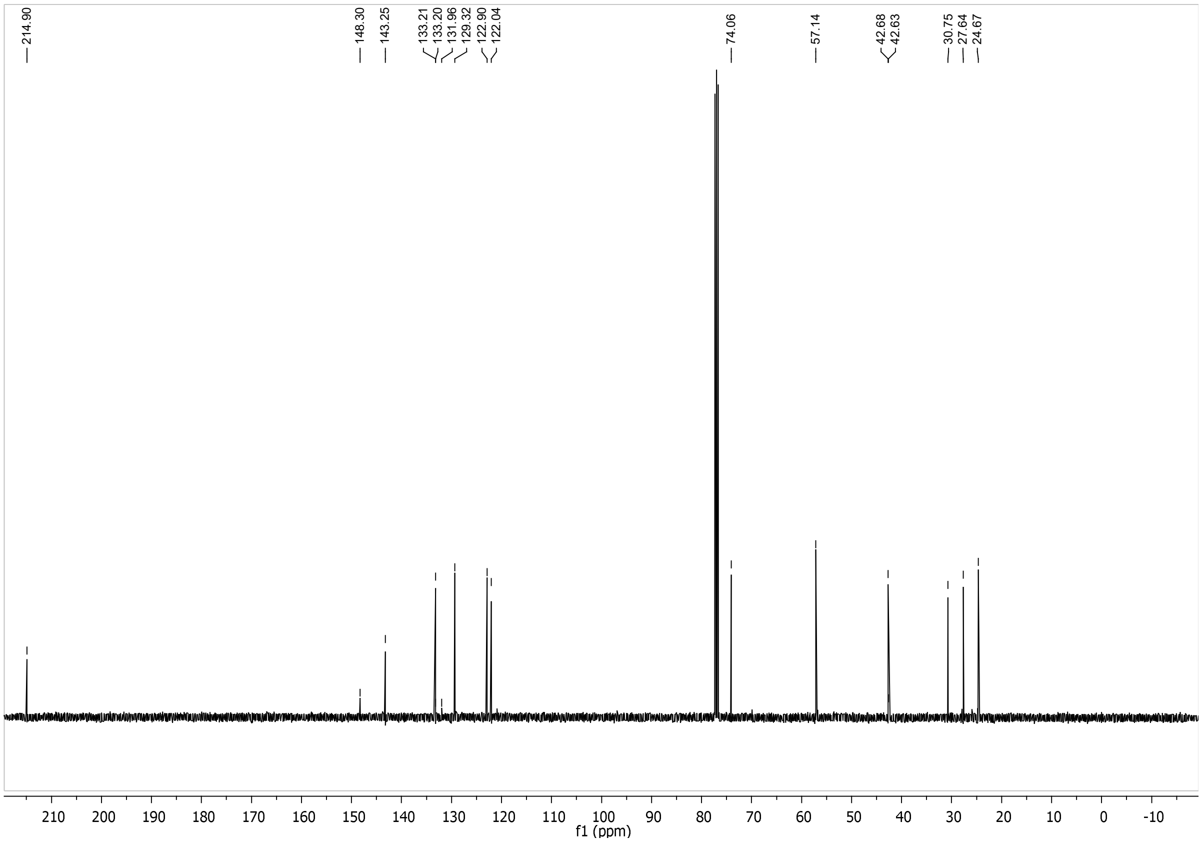

(S)-2-((R)-(2-chlorophenyl)(hydroxy)methyl)cyclohexan-1-one (3c)

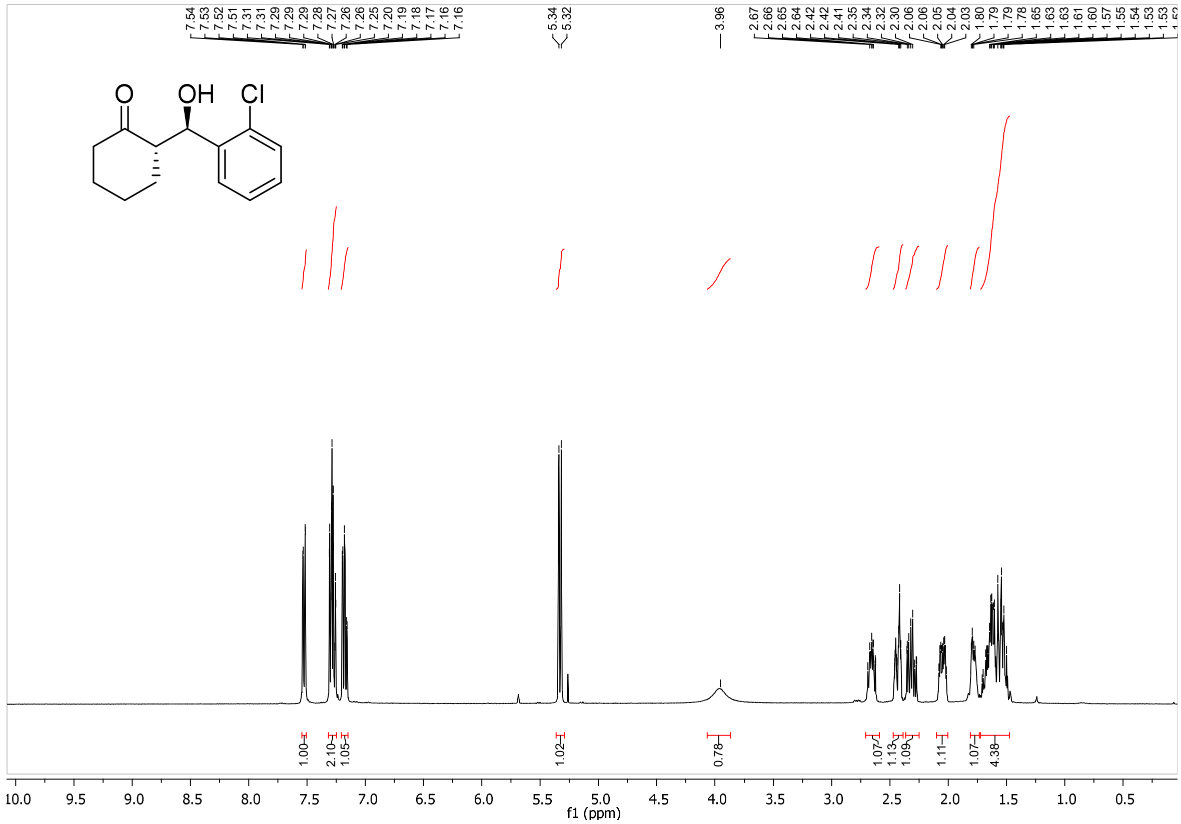

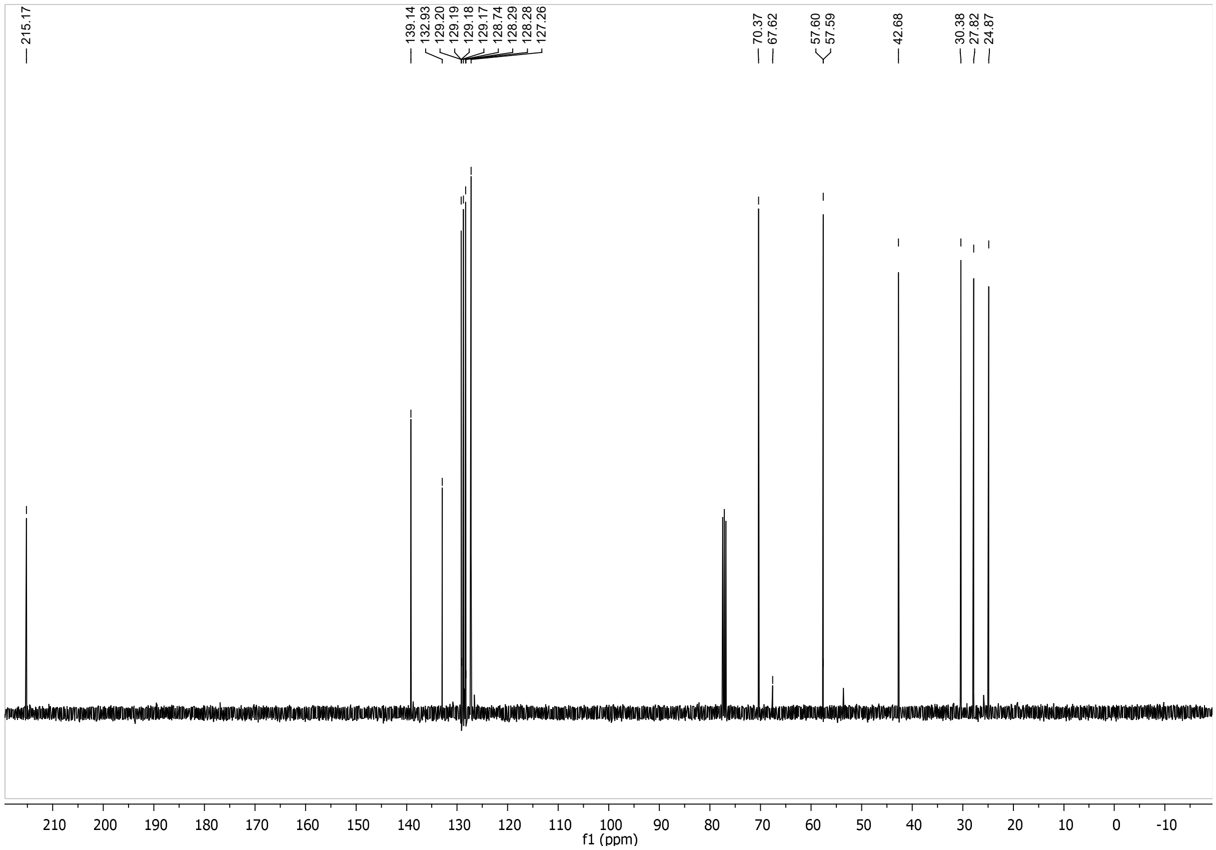

(S)-2-((R)-(4-bromophenyl)(hydroxy)methyl)cyclohexan-1-one (3d)

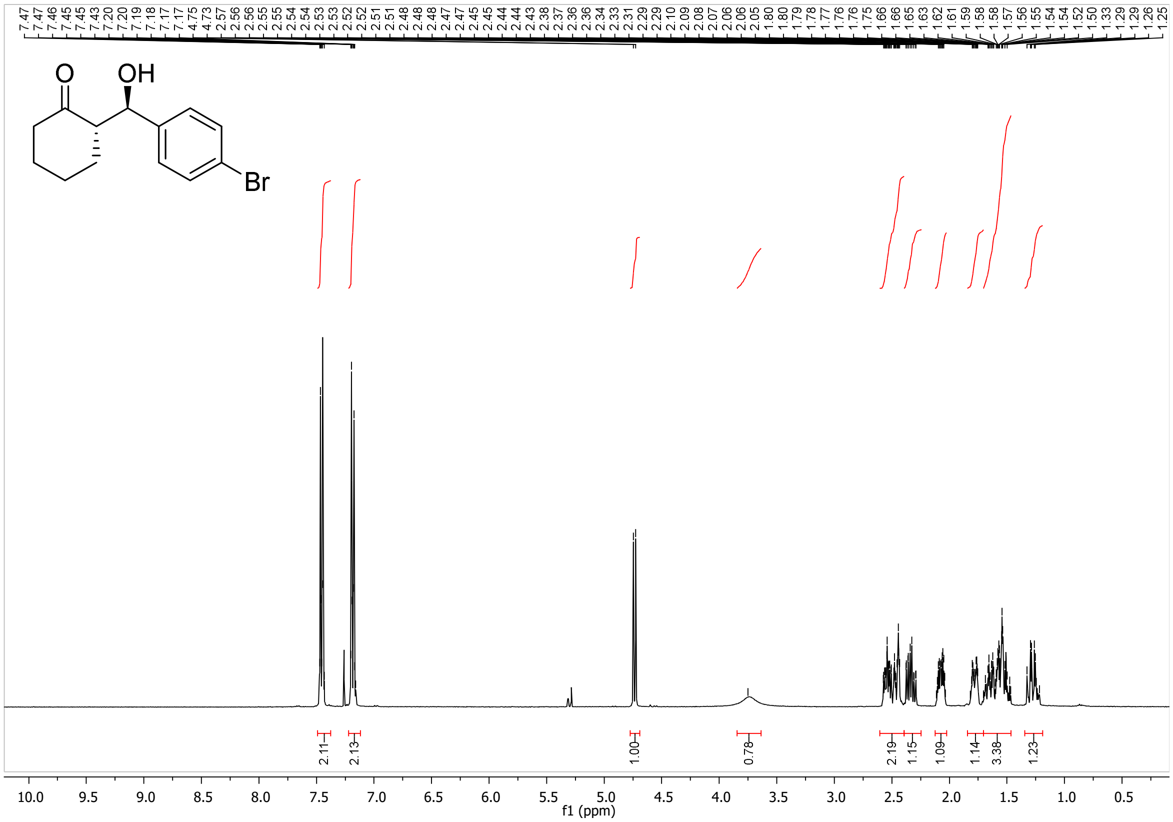

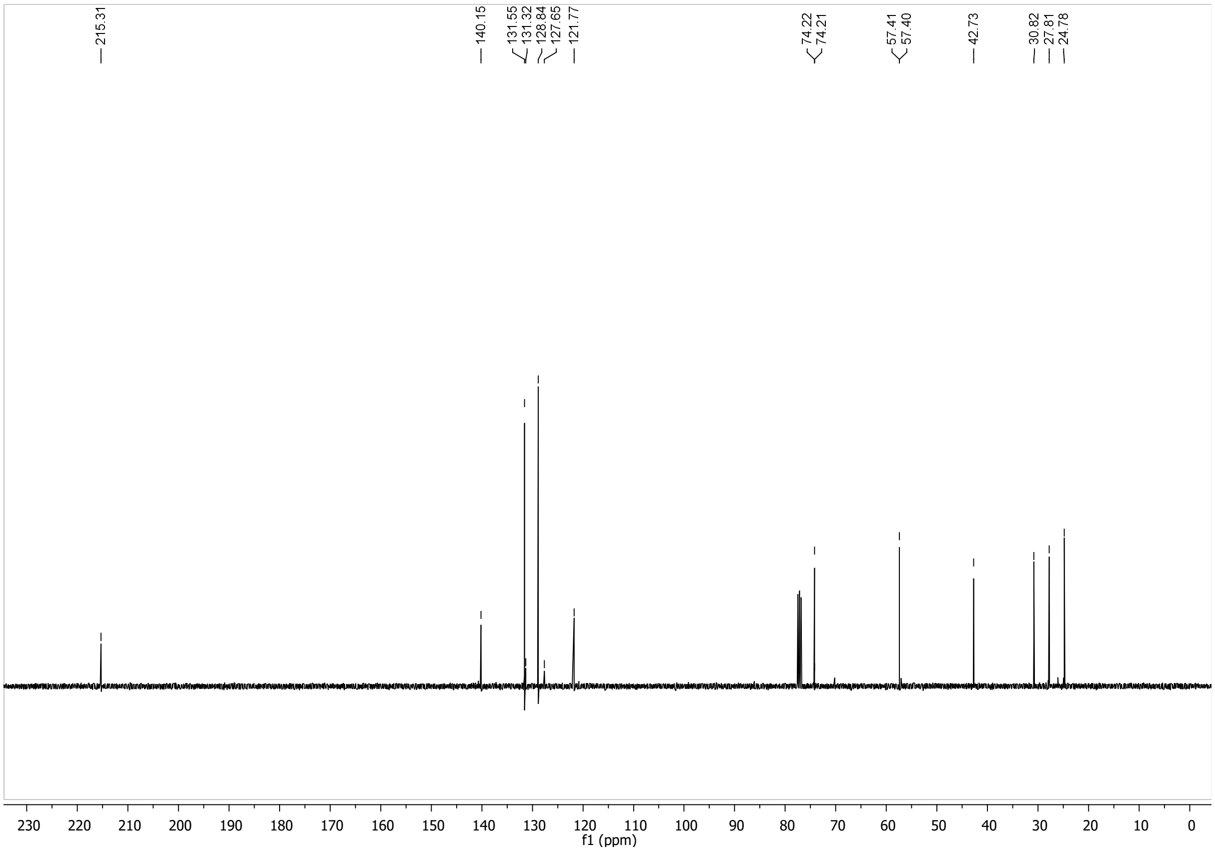

HPLC DATA

(S)-2-((R)-hydroxy(4-nitrophenyl)methyl)cyclohexan-1-one (X)

SHIMADZU LabSolutions **Analysis Report**

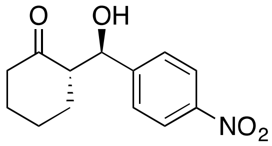

<Sample Information>

Sample Name	: CD65		
Sample ID	: CD65		
Data Filename	: CD65.lcd		
Method Filename	: H-iPrOH_95-5_1mlmin.lcm		
Batch Filename	:		
Vial #	: 1-1	Sample Type	: Unknown
Injection Volume	: 1 uL		
Date Acquired	: 17/03/2022 17:09:44	Acquired by	: System Administrator
Date Processed	: 17/03/2022 17:58:10	Processed by	: System Administrator

<Chromatogram>

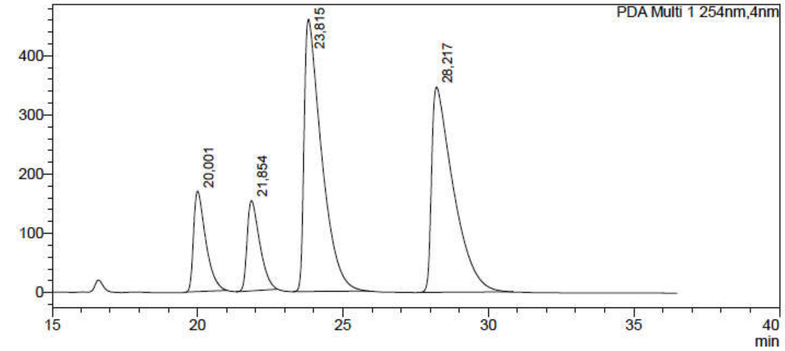

<Peak Table>

PDA Ch1 254nm

Peak#	Ret. Time	Area	Height	Area%
1	20,001	4950415	169548	10,548
2	21,854	4598570	152293	9,799
3	23,815	19254976	459733	41,029
4	28,217	18126578	346597	38,624
Total		46930538	1128171	100,000

<Sample Information>

Sample Name	: CD 103		
Sample ID	: CD 103		
Data Filename	: CD 103.lcd		
Method Filename	: Hex_iPro_95_5.lcm		
Batch Filename	:		
Vial #	: 1-1	Sample Type	: Unknown
Injection Volume	: 1 uL		
Date Acquired	: 30/06/2022 14:51:05	Acquired by	: System Administrator
Date Processed	: 01/07/2022 13:31:13	Processed by	: System Administrator

<Chromatogram>

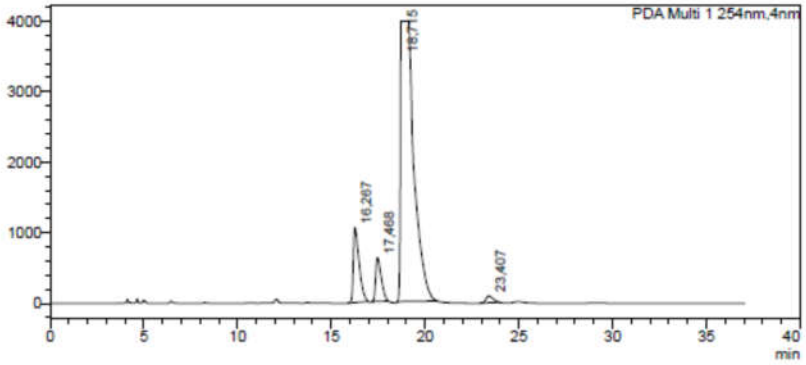

<Peak Table>

PDA Ch1 254nm

Peak#	Ret. Time	Area	Height	Area%
1	16,267	22864421	1059916	9,767
2	17,468	12910651	622412	5,515
3	18,715	195899067	3975108	83,680
4	23,407	2430270	94067	1,038
Total		234104409	5751503	100,000

(S)-2-((R)-hydroxy(3-nitrophenyl)methyl)cyclohexan-1-one (3b)

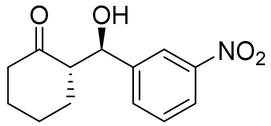

<Sample Information>

Sample Name	: CD 113 con C4D6 0,5		
Sample ID	: CD 113 con C4D6 0,5		
Data Filename	: CD 113 con C4D6 0,5.lcd		
Method Filename	: Hex_iPro_95_5.lcm		
Batch Filename	:		
Vial #	: 1-1	Sample Type	: Unknown
Injection Volume	: 1 uL		
Date Acquired	: 20/07/2022 13:17:02	Acquired by	: System Administrator
Date Processed	: 20/07/2022 17:47:50	Processed by	: System Administrator

<Chromatogram>

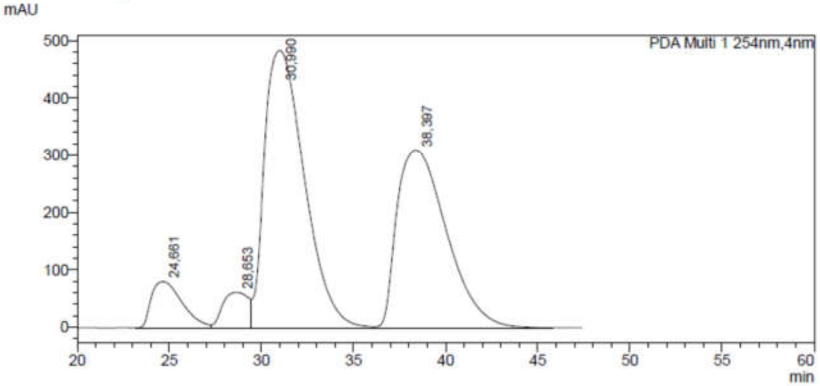

<Peak Table>

PDA Ch1 254nm

Peak#	Ret. Time	Area	Height	Area%
1	24,661	9396329	80658	6,413
2	28,653	5950630	61819	4,061
3	30,990	73076041	483790	49,874
4	38,397	58097332	309518	39,651
Total		146520332	935785	100,000

<Sample Information>

Sample Name	: CD 112 0,5 p2		
Sample ID	: CD 112 0,5 p2		
Data Filename	: CD 112 chiral 0,5 p2.lcd		
Method Filename	: Hex_iPro_95_5.lcm		
Batch Filename	:		
Vial #	: 1-1	Sample Type	: Unknown
Injection Volume	: 1 uL		
Date Acquired	: 20/07/2022 15:12:14	Acquired by	: System Administrator
Date Processed	: 20/07/2022 15:57:06	Processed by	: System Administrator

<Chromatogram>

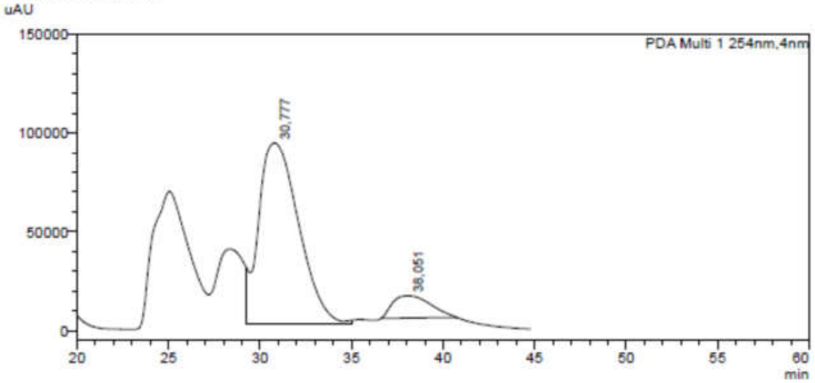

<Peak Table>

PDA Ch1 254nm

Peak#	Ret. Time	Area	Height	Area%
1	30,777	14003083	91706	89,572
2	38,051	1630231	11252	10,428
Total		15633313	102958	100,000

(S)-2-((R)-(2-chlorophenyl)(hydroxy)methyl)cyclohexan-1-one (3c)

<Sample Information>

Sample Name	: CD115		
Sample ID	: CD115		
Data Filename	: CD115.lcd		
Method Filename	: Hex_iPro_95_5.lcm		
Batch Filename	:		
Vial #	: 1-1	Sample Type	: Unknown
Injection Volume	: 1 uL		
Date Acquired	: 18/07/2022 15:04:15	Acquired by	: System Administrator
Date Processed	: 18/07/2022 15:40:59	Processed by	: System Administrator

<Chromatogram>

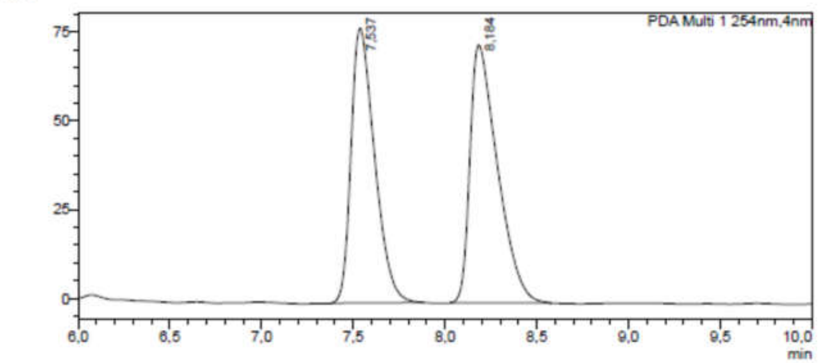

<Peak Table>

PDA Ch1 254nm

Peak#	Ret. Time	Area	Height	Area%
1	7,537	692475	77270	47,779
2	8,184	756865	72722	52,221
Total		1449339	149992	100,000

<Sample Information>

Sample Name	: CD114		
Sample ID	: CD114		
Data Filename	: CD114.lcd		
Method Filename	: Hex_iPro_95_5.lcm		
Batch Filename	:		
Vial #	: 1-1	Sample Type	: Unknown
Injection Volume	: 1 uL		
Date Acquired	: 18/07/2022 15:22:09	Acquired by	: System Administrator
Date Processed	: 18/07/2022 15:39:00	Processed by	: System Administrator

<Chromatogram>

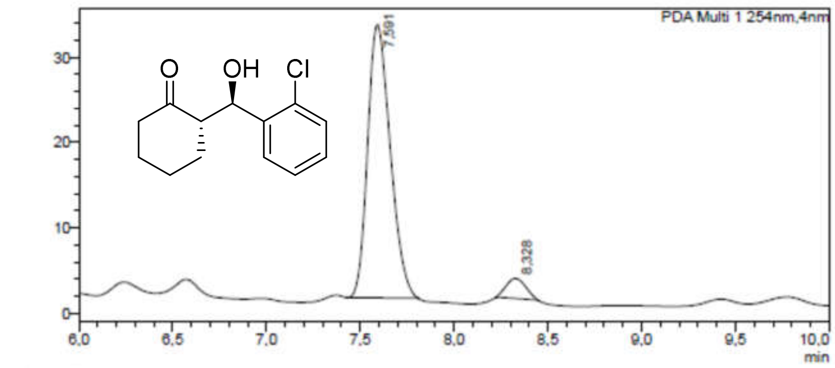

<Peak Table>

PDA Ch1 254nm

Peak#	Ret. Time	Area	Height	Area%
1	7,591	268441	32012	94,017
2	8,328	17083	2350	5,983
Total		285523	34362	100,000

(S)-2-((R)-(4-bromophenyl)(hydroxy)methyl)cyclohexan-1-one (3d)

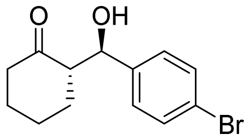

<Sample Information>

Sample Name	: CD 120COL		
Sample ID	: CD 120COL		
Data Filename	: CD 120COL.lcd		
Method Filename	: Hex_iPro_95_5.lcm		
Batch Filename	:		
Vial #	: 1-1	Sample Type	: Unknown
Injection Volume	: 1 uL		
Date Acquired	: 25/07/2022 13:23:58	Acquired by	: System Administrator
Date Processed	: 25/07/2022 15:11:34	Processed by	: System Administrator

<Chromatogram>

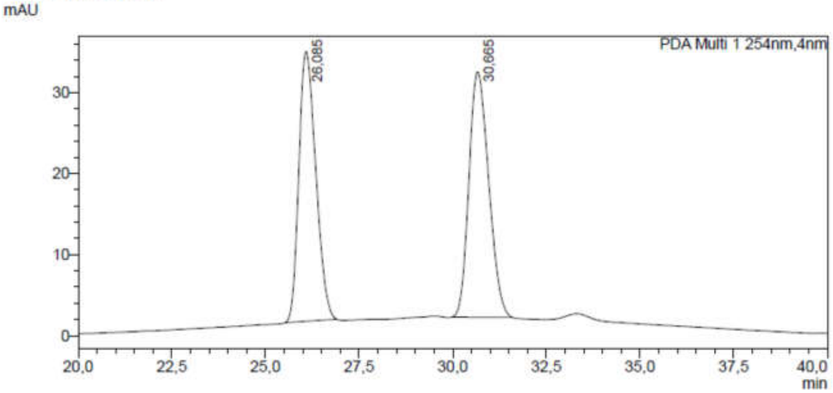

<Peak Table>

PDA Ch1 254nm

Peak#	Ret. Time	Area	Height	Area%
1	26,085	1046305	33285	48,548
2	30,665	1108904	30281	51,452
Total		2155209	63566	100,000

<Sample Information>

Sample Name	: CD 119EST chir		
Sample ID	: CD 119EST chir		
Data Filename	: CD 119EST chir.lcd		
Method Filename	: Hex_iPro_95_5.lcm		
Batch Filename	:		
Vial #	: 1-1	Sample Type	: Unknown
Injection Volume	: 1 uL		
Date Acquired	: 25/07/2022 14:06:54	Acquired by	: System Administrator
Date Processed	: 25/07/2022 15:15:26	Processed by	: System Administrator

<Chromatogram>

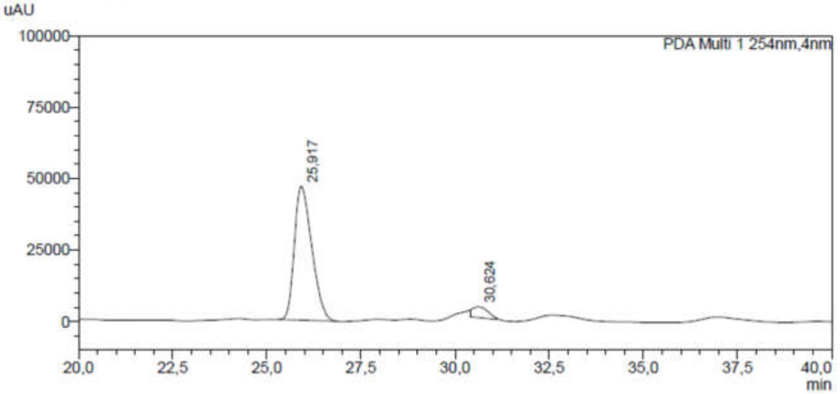

<Peak Table>

PDA Ch1 254nm

Peak#	Ret. Time	Area	Height	Area%
1	25,917	1504360	46681	93,324
2	30,624	107616	3777	6,676
Total		1611976	50458	100,000
